# National Trends and Policy Impacts on Provision of Home Medicines Reviews and Residential Medication Management Reviews in Older Australians, 2009–2019

**DOI:** 10.3390/ijerph18189898

**Published:** 2021-09-20

**Authors:** Janet K. Sluggett, Luke R. Collier, Jonathan D. Bartholomaeus, Maria C. Inacio, Steve L. Wesselingh, Gillian E. Caughey

**Affiliations:** 1 UniSA Allied Health and Human Performance, University of South Australia, Adelaide, SA 5001, Australia; maria.inacio@sahmri.com (M.C.I.); gillian.caughey@sahmri.com (G.E.C.); 2 Registry of Senior Australians (ROSA), Healthy Ageing Research Consortium, South Australian Health and Medical Research Institute, Adelaide, SA 5001, Australia; luke.collier@sahmri.com (L.R.C.); jonathan.bartholomaeus@adelaide.edu.au (J.D.B.); steve.wesselingh@sahmri.com (S.L.W.); 3 Centre for Medicine Use and Safety, Faculty of Pharmacy and Pharmaceutical Sciences, Monash University, Parkville, VIC 3052, Australia; 4 School of Psychology, The University of Adelaide, Adelaide, SA 5005, Australia

**Keywords:** nursing homes, long-term care, residential facilities, residential aged care, medication review, medication therapy management, Australia

## Abstract

Comprehensive medicines reviews such as Home Medicines Review (HMR) and Residential Medication Management Review (RMMR) can resolve medicines-related problems. Changes to Australia’s longstanding HMR and RMMR programs were implemented between 2011 and 2014. This study examined trends in HMR and RMMR provision among older Australians during 2009–2019 and determined the impact of program changes on service provision. Monthly rates of general medical practitioner (GP) HMR claims per 1000 people aged ≥65 years and RMMR claims per 1000 older residents of aged care facilities were determined using publicly available data. Interrupted time series analysis was conducted to examine changes coinciding with dates of program changes. In January 2009, monthly HMR and RMMR rates were 0.80/1000 older people and 20.17/1000 older residents, respectively. Small monthly increases occurred thereafter, with 1.89 HMRs/1000 and 34.73 RMMRs/1000 provided in February 2014. In March 2014, immediate decreases of –0.32 (95%CI –0.52 to –0.11) HMRs/1000 and –12.80 (95%CI –15.22 to –10.37) RMMRs/1000 were observed. There were 1.07 HMRs/1000 and 35.36 RMMRs/1000 provided in December 2019. In conclusion, HMR and RMMR program changes in March 2014 restricted access to subsidized medicines reviews and were associated with marked decreases in service provision. The low levels of HMR and RMMR provision observed do not represent a proactive approach to medicines safety and effectiveness among older Australians.

## 1. Introduction

Older people are at increased risk of medicines-related harm. Strategies are needed to address the harm arising from medicines use that contributes to an estimated 250,000 hospital admissions annually in Australia [1]. Comprehensive medicines reviews can improve medicines safety and efficacy in older people. Home Medicines Review (HMR) and Residential Medication Management Review (RMMR) are flagship Australian Government-subsidized comprehensive medicines review programs that involve an accredited pharmacist visiting the patient’s home or residential aged care facility (RACF) to obtain a best possible medicines history, provide education, and identify medicines-related problems [2]. The pharmacist provides the referring general medical practitioner (GP) with a report describing how to address any medicines-related problems, which the GP uses to prepare a medicines management plan with the patient. Similar models exist in other countries [2,3]. Broad and consistent implementation of HMRs and RMMRs, which identify an average of three to four medicines-related problems per review [3,4], has been recommended nationally to reduce harms arising from inappropriate polypharmacy in older Australians [5].

The HMR and RMMR programs have operated for over 20 years, with major changes to program funding rules between 2011 and 2014. Examination of claims data suggests these program changes coincided with changes in service provision [6,7,8] but has not been investigated in older people specifically. Understanding the impacts of program changes is necessary to inform future policy responses to the findings of the recent Royal Commission into Aged Care Quality and Safety, Australia’s response to the World Health Organization’s *Medication Without Harm* and Australia’s new national health priority area ‘Quality Use of Medicines and Medicines Safety’ [5,9]. This study investigated trends in HMR and RMMR provision among older Australians between 2009 and 2019 and determined the impact of program changes between 2011 and 2014 on HMR/RMMR provision.

## 2. Materials and Methods

### 2.1. Study Design and Data Sources

A repeated, cross-sectional study was conducted using publicly available Medicare Benefits Schedule (MBS) processing data, Australian Bureau of Statistics data, and Australian Institute of Health and Welfare (AIHW) GEN aged care data. MBS program rules require GPs to lodge HMR/RMMR claims after the medicines management plan is discussed with the patient. The total monthly number of GP claims processed for HMRs (MBS item codes 900, 245) and RMMRs (903, 249) between January 2009 and December 2019 for individuals aged ≥65 years was extracted from the Services Australia website [10]. Quarterly national population projections obtained from the Australian Bureau of Statistics [11] were used to determine monthly HMR rates per 1000 older persons. Yearly estimates of people aged ≥65 years using permanent residential aged care on 30 June each year were obtained from AIHW GEN website [12] and this denominator was applied to determine monthly RMMR rates per 1000 older residents. Ethical approval was not sought as publicly available, de-identified data were analyzed.

### 2.2. Statistical Analysis

Trends were stratified by age group (65–74, 75–84, and ≥85 years) and sex. Interrupted time series analysis was conducted using seasonal autoregressive integrated moving average (ARIMA) modelling [13]. Program changes for HMRs (October 2011, March 2013, March 2014) and RMMRs (October 2011, March 2014) (Table 1) were modelled as change points. Optimal seasonal ARIMA models were selected based on inspection of the model residuals and fit statistics computed via *auto.arima* in R V4.0.2 (R Foundation for Statistical Computing, Vienna, Austria) [13]. Step (i.e., change in level immediately following a program change) and ramp (i.e., gradual slope change relative to the previous period) functions were included at the date of each program change to estimate both immediate impacts and changes over time. The counterfactual forecasted trend with 80% and 95% prediction intervals was undertaken using *forecast* in R.

## 3. Results

In January 2009, there were 2,869,028 individuals (54.5% women) aged ≥65 years nationally, with 2292 GP claims for HMRs. There were 3073 GP claims for RMMRs in January 2009 and there were 152,376 older residents (71.8% women) included in the RACF denominator in 2009. The populations of interest increased to 4,107,641 individuals and 177,098 residents in December 2019, with 4405 HMRs and 6262 RMMRs provided. 

**Figure 1:**
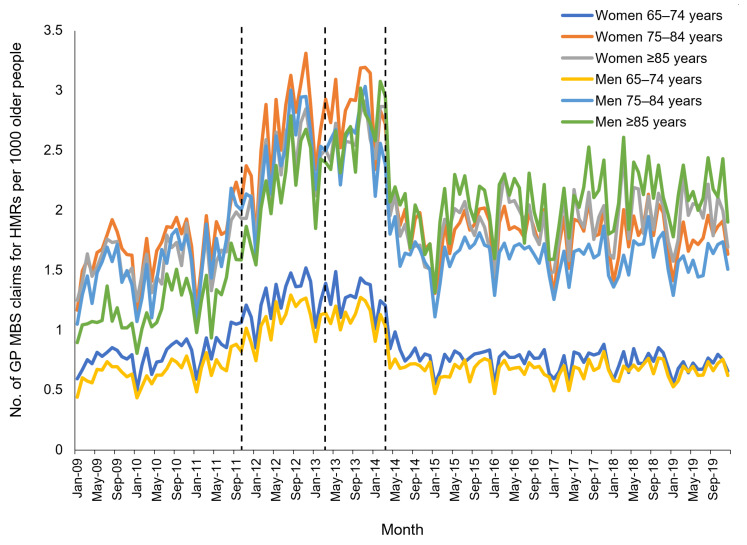
Monthly rate of GP claims for Home Medicines Reviews (HMRs) per 1000 older persons between 2009 and 2019, stratified by age group and sex. *Dashed lines indicate dates of program changes*.

**Figure 2:**
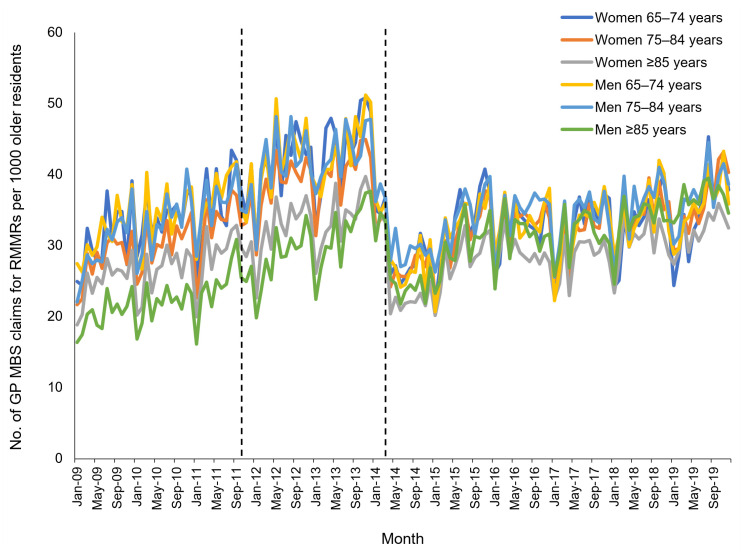
Monthly rate of GP claims for Residential Medication Management Reviews (RMMRs) per 1000 older residents of aged care facilities between 2009 and 2019, stratified by age group and sex. *Dashed lines indicate dates of program changes*.

Monthly rates of HMRs and RMMRs per 1000 older people by age group and sex are shown in Figure 1 and Figure 2. Across all age groups, HMR provision increased at a greater rate after October 2011, then sharply declined between March 2014 and January 2015, remaining steady thereafter (Figure 1). HMR rates were lower in individuals aged 65–74 years compared to those aged ≥75 years.

RMMR provision steadily increased across all age groups from January 2009; however, decreased provision was noted after March 2014 (Figure 2). Provision slowly recovered between 2015 and 2019 but did not reach pre-2014 levels by December 2019.

### 3.1. Impact of Program Changes on HMR Provision

**Figure 3:**
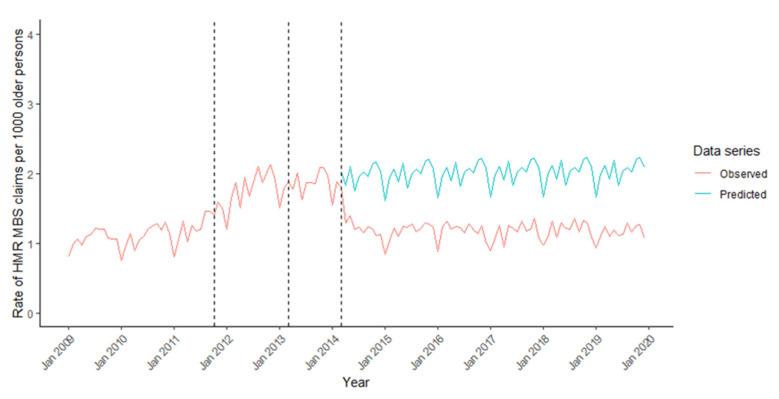
Monthly rate of GP claims for Home Medicines Reviews per 1000 older persons between 2009 and 2019, and counterfactual forecast in the absence of changes to program rules in 2014. *Dashed lines indicate dates of program rule changes*.

Trends in HMR provision in older people and counterfactual forecasting of HMR provision in the absence of 2014 program changes are shown in Figure 3.

In January 2009, 0.80/1000 older people received HMRs, increasing to 1.46/1000 in September 2011. There was a small monthly gradual increase in HMR provision after October 2011 compared to the previous period (0.03, 95%CI 0.01 to 0.06) (Table 2). There was no significant change in the rate of HMR provision after March 2013. However, an immediate reduction in HMR provision was observed in March 2014, with −0.32 (95%CI −0.52 to −0.11) fewer HMRs/1000, with low rates of provision sustained thereafter.

Comparison between observed and forecasted trends (i.e., without the March 2014 program changes) suggested there were 41.3% fewer HMRs/1000 per month than predicted 12 months later (i.e., March 2015) (Figure S1).

### 3.2. Impact of Program Changes on RMMR Provision

**Figure 4:**
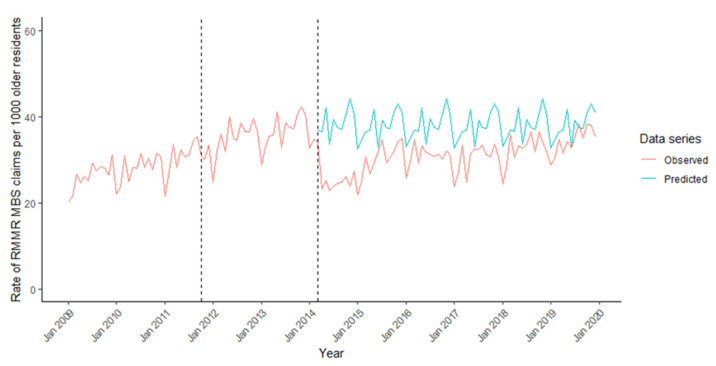
Monthly rate of GP claims for Residential Medication Management Reviews (RMMRs) per 1000 older residents of aged care facilities between 2009 and 2019, and counterfactual forecast in the absence of changes to program rules in 2014. *Dashed lines indicate dates of program rule changes*.

Figure 4 shows that RMMR provision increased from 20.17 to 35.24 RMMRs/1000 between January 2009 and September 2011. RMMR provision continued to increase at a similar rate between October 2011 and February 2014 relative to the previous period (0.05, 95%CI −0.12 to 0.21) (Table 2). There was an immediate decrease in RMMR provision in March 2014 (−12.80, 95%CI −15.22 to −10.37). Between April 2014 and December 2019, RMMR provision increased from 23.29 to 35.36 RMMRs/1000, which was similar to the monthly rate of increase prior to March 2014 (−0.15, 95%CI −0.27 to −0.02).

In March 2015, there were 15.7% fewer RMMRs/1000 residents per month than predicted had the program rules not changed in March 2014 (Figure S2).

## 4. Discussion

RMMR and HMR provision is low in older Australians and was significantly impacted by policy changes during 2011–2014. Variation in HMR and RMMR provision by age and sex was observed. Our results are consistent with recent analyses showing significant national variation and overall low uptake of HMRs and RMMRs [14,15,16,17,18,19]. Du et al. examined GP MBS claims for HMRs among individuals participating in the 45 and Up study between 2009 and 2014 and found that 4.7% of the cohort received at least one HMR during this period, with higher rates of service provision noted with increasing age [17]. An Australian study examining trends in HMR provision among community-dwelling veterans between 2001 and 2016 reported an increase in the monthly HMR rate from 0.2 HMRs per 1000 individuals in November 2001 to 4.5 per 1000 in March 2012, reducing to 2 HMRs per 1000 individuals in December 2016 [18]. Recent analyses of national claims data found that at least one HMR or RMMR was provided to 3.9% of individuals aged 75–84 years and 10.2% of those aged ≥85 years in 2018–19 [15]. National studies examining RMMR provision have identified that only one in five individuals entering RACFs between 2012 and 2015 received an RMMR within 3 months of entry [14,16], while annual age- and sex-adjusted rates of HMR/RMMR provision among permanent residents of RACFs increased from 18.3% in 2006 to 45.5% in 2015 [19]. Findings of the present study, together with existing HMR and RMMR research, show many older people continue to miss out on these valuable services.

Overall, GP claiming for HMRs and RMMRs continued to increase in older people following program changes in October 2011 that enabled referrals to be sent directly to accredited pharmacists and the removal of pharmacist-initiated RMMRs. While the changes in 2011 may have in part facilitated more collaborative medicines reviews, examination of crude pharmacist claims data suggest these changes led to an overall decrease in RMMR provision by pharmacists resulting from the removal of pharmacist-initiated RMMRs [7]. Pharmacists claim for HMRs and RMMRs via a different mechanism to GPs. Comparison of the crude number of RMMR claims submitted by pharmacists and GPs in 2009, when pharmacist-initiated reviews were permitted, suggests four in 10 RMMRs were collaborative [10,20]. In our study, HMR provision plateaued following the reinforcement of program rules in March 2013 in response to concern about program expenditure and HMRs conducted outside the patient’s home (comprising 12% of all pharmacist HMR claims) [7]. In March 2014, marked and sustained decreases in the rates of HMR and RMMR claims by GPs for older people were observed after the minimum time frame between repeat pharmacist claims was extended and caps of 20 HMRs per month per provider were enforced. The rate of HMR provision did not recover after the changes to the program rules in 2014, with little difference between the monthly rate of GP claims for HMRs between the start and the end of the study period. RMMR provision slowly recovered between April 2014 and December 2019 but did not reach pre-2014 rates of provision. These findings are concerning, given increases in resident age, care needs, multimorbidity, and high-risk medicines utilization in RACFs during this period [19] and recent national recommendations to address medicines safety through broad and consistent access to these programs for older people [5,9].

Overall, program changes in 2014 were found to be a major barrier to HMR and RMMR provision in older Australians. More needs to be done to improve HMR/RMMR uptake, service delivery, and uptake of pharmacist recommendations. Strategies could include consumer and GP education, targeted feedback to GPs that identifies patients who may benefit from HMRs, standardized templates, peer assessment of reports, and better utilization of digital systems and decision support tools [5,15,18,21,22]. Program rules could be changed to, in part, facilitate greater flexibility and include strategies such as referral by nurse practitioners, providing HMR visits outside the patient’s home (e.g., in general practices), removing caps on monthly HMR provision, quality indicators, and greater involvement of RACF medication advisory committees [6,14,15,18,21,23]. Recent recommendations from Australia’s MBS Taskforce to establish MBS items for remunerating pharmacists and other non-medical health professionals for case conference participation have not yet been implemented [24]. Additionally, data on long-term outcomes, cost effectiveness, and patient-reported measures are needed to ensure individuals most likely to benefit from medicines reviews are targeted and to inform program refinements [3,4,6,14].

We suggest the proposed changes need to incorporate evidence that good working relationships between health professionals, face-to-face discussions, and positive organizational culture are enablers to high-quality medicines reviews [21,25,26]. Program changes could also incorporate new models of care for medicines reviews. For example, post-discharge medicines reviews by integrated general practice pharmacists can reduce hospital readmissions and emergency department presentations [27]. Provision of HMRs at the time of an aged care eligibility assessment can resolve medicines-related problems [28], while integration of pharmacists within RACFs improves medication administration practices [29]. Australia’s recent Royal Commission into Aged Care Quality and Safety recommended increased access to RMMRs and pharmacist services in RACFs [9]. However, system changes will be required to support enhanced and proactive models of care for older people. HMR provision was found to be three to six times lower than population need in 2018–19, with an estimated $91 million of additional government expenditure necessary to address this gap [6]. The number of pharmacists completing accreditation to provide medicines reviews has stabilized since 2014 and substantial workforce changes will be required to deliver extra services [6,30]. Systems change will require substantial investment to cover additional costs of HMR/RMMR provision or other new services, workforce training and education, and research to enhance services and comprehensively monitor outcomes.

This nationally representative study captured all GP claims for HMRs and RMMRs processed via the MBS; however, individual-level information about recipients and service quality was not available. ARIMA modelling enabled us to account for non-stationarity, seasonality, and autocorrelation [13], but there was less certainty in forecasted trends (i.e., in the absence of the policy changes) towards the end of the study period. Although program changes coincided with changes in MBS claiming rates, we cannot rule out other contributors. Immediate impacts of program changes (i.e., change in level) may be underestimated as month-of-claim processing (not month-of-claim submission) was analyzed, and the trends underestimate services provided by pharmacists. In 2019, more pharmacist claims for medicines reviews (95,491 HMRs, 124,422 RMMRs) were reimbursed than GP claims (71,992 HMRs, 75,647 RMMRs) [10,20]. Analyzing MBS claims may, therefore, underestimate the number of reports prepared by pharmacists, who claim after sending their report to the GP [14,16]. The denominator for permanent residents of RACFs was only available on an annual basis rather than quarterly. While there is a 25–30% turnover in residents annually, RACFs operate at an approximate 90% occupancy rate [31], so our denominator likely reflects the numbers of permanent residents in RACFs each month. RMMR provision may be slightly overestimated as RMMRs can occasionally be provided to individuals receiving transition care, who were not included in our denominator. On 30 June 2019, there were 3603 individuals receiving transition care and just under half (46%) accessed some or all services from an RACF [31,32]. The impact of program changes in early 2020 [33] that increased the monthly cap to 30 HMRs and enabled additional specialist physicians to refer, provision of pharmacist follow-up services, and temporary delivery via telehealth during the COVID-19 pandemic requires evaluation.

## 5. Conclusions

Changes to HMR and RMMR program rules in March 2014 that restricted access were associated with marked decreases in the rates of GP HMR and RMMR claims for older Australians. Low rates of HMR provision persisted until 2019, while trends in RMMR provision recovered slowly between 2015 and 2019. Australia’s medicines review program is one of few subsidized, national services to support quality use of medicines in older people. Yet, the low levels of HMR and RMMR provision observed in this study do not represent a proactive approach to medicines safety and effectiveness. Systems changes are needed to address these gaps among older Australians and move the needle closer towards quality use of medicines.

## Figures and Tables

**Table 1:** Summary of changes to the HMR and RMMR programs during 2009 and 2019 [7,8].

Home Medicines Review (HMR)	Residential Medication Management Review (RMMR)
***Prior to October 2011***	
All HMRs were collaborative (i.e., in conjunction with the individual’s usual GP). HMR claims could only be lodged via an approved community pharmacy. Pharmacies could lodge one HMR claim for an individual patient in a 12-month period. MBS criteria allowed GPs to refer for the service every 12 months if clinically indicated.	RMMRs could be pharmacist-initiated or collaborative. Claims could only be lodged via an approved community pharmacy. Pharmacies could lodge one RMMR claim for an individual patient in a 12-month period. MBS criteria allowed GPs to refer for the service every 12 months if clinically indicated. RMMR services were integrated with QUM services (i.e., facility-level medicines advisory, educational and continuous improvement activities).
***October 2011*** ^1^	
HMR referrals could be directed to the patient’s preferred pharmacy or accredited pharmacist (i.e., direct referral pathway).	All RMMRs needed to be collaborative with the GP (i.e., required a GP referral). Pharmacist-initiated RMMRs could only be provided in exceptional circumstances and required prior approval from the Department of Health. RMMR referrals could be directed to an approved community pharmacy or an accredited pharmacist. QUM services were separated from RMMR services.
***15 March 2013***	
Reinforcement that HMRs must be conducted by an accredited pharmacist and provided in the recipient’s own home (unless prior approval obtained for an alternative location). Pharmacist claims must be lodged within 30 days of providing the service.	
***1 March 2014***	
Maximum number of HMRs that a pharmacist could undertake was capped at 20 per month. The cap did not apply to RMMRs. Pharmacists required to conduct the HMR within 90 days of referral date. Minimum timeframe of 24 months between pharmacist claims for HMRs for the same individual unless an earlier referral was provided by the GP (no changes to MBS criteria for GP claiming).	Pharmacists required to conduct the RMMR within 90 days of referral date. Pharmacist claims must be lodged within 30 days of providing the service. Minimum time frame of 24 months between pharmacist claims for RMMRs for the same individual unless an earlier referral was provided by the GP (no changes to MBS criteria for GP claiming).
***1 July 2018*** ^2^	
New MBS item number 245 introduced as part of the Australian Government’s Stronger Rural Health Strategy to enable new non-vocationally registered GPs to claim for HMRs.	New MBS item number 249 introduced as part of the Australian Government’s Stronger Rural Health Strategy to enable new non-vocationally registered GPs to claim for RMMRs.

^1^ The MedsCheck program was introduced in community pharmacies on 1 July 2012. ^2^ The impact of this change was not analyzed in the interrupted time series analysis as small numbers of GP MBS claims were lodged for MBS items 245 or 249 among patients aged 65 years and over in 2019 (n = 1143 claims overall). GP, general medical practitioner; HMR, Home Medicines Review; MBS, Medicare Benefits Schedule; QUM, Quality Use of Medicines; RMMR, Residential Medication Management Review.

**Table 2:** Step and ramp estimates from ARIMA models.

Month of Program Change.	Type of Change ^1,2^	Estimate (95% CI)	*p*-Value
**Home Medicines Reviews**			
October 2011	Step	−0.01 (−0.20, 0.18)	0.90
	Ramp	0.03 (0.01, 0.06)	0.04
March 2013	Step	0.04 (−0.16, 0.23)	0.72
	Ramp	−0.03 (−0.09, 0.04)	0.42
March 2014	Step	−0.32 (−0.52, −0.11)	<0.01
	Ramp	−0.01 (−0.06, 0.04)	0.70
**Residential Medication Management Reviews**			
October 2011	Step	−0.54 (−3.30, 2.23)	0.70
	Ramp	0.05 (−0.12, 0.21)	0.58
March 2014	Step	−12.80 (−15.22, −10.37)	<0.01
	Ramp	−0.15 (−0.27, −0.02)	0.02

^1^ Step = change in level immediately following a program change. ^2^ Ramp = gradual slope change relative to the slope in the period prior to the program change. ARIMA, Auto-Regressive Integrated Moving Averages; CI, confidence interval.

## Data Availability

This study analyzed publicly available Medicare Benefits Schedule (MBS) processing data and Australian Bureau of Statistics (ABS) and Australian Institute of Health and Welfare (AIHW) GEN aged care data.

## Supplementary Materials

The following are available online at https://www.mdpi.com/article/10.3390/ijerph18189898/s1. Figure S1: Monthly rate of Home Medicines Review (HMR) claims on the Medicare Benefits Schedule per 1000 older persons between 2009 and 2019, and counterfactual forecast in the absence of changes to program rules in 2014. Figure S2: Monthly rate of Residential Medication Management Review (RMMR) claims on the Medicare Benefits Schedule per 1000 older residents of aged care facilities between 2009 and 2019, and counterfactual forecast in the absence of changes to program rules in 2014.
