# Supplementary material for: National Trends and Policy Impacts on Provision of Home Medicines Reviews and Residential Medication Management Reviews in Older Australians, 2009–2019
Source: Int J Environ Res Public Health. 2021 Sep 20;18(18):9898. doi: 10.3390/ijerph18189898 (PMC8467825; doi:10.3390/ijerph18189898)
Supplement: Supplementary file 1 [file ijerph-18-09898-s001.zip › ijerph-1382026-supplementary.pdf]

Figure S1. Monthly rate of Home Medicines Review (HMR) claims on the Medicare Benefits Schedule per 1000 older persons between 2009 and 2019, and counterfactual forecast in the absence of changes to program rules in 2014.

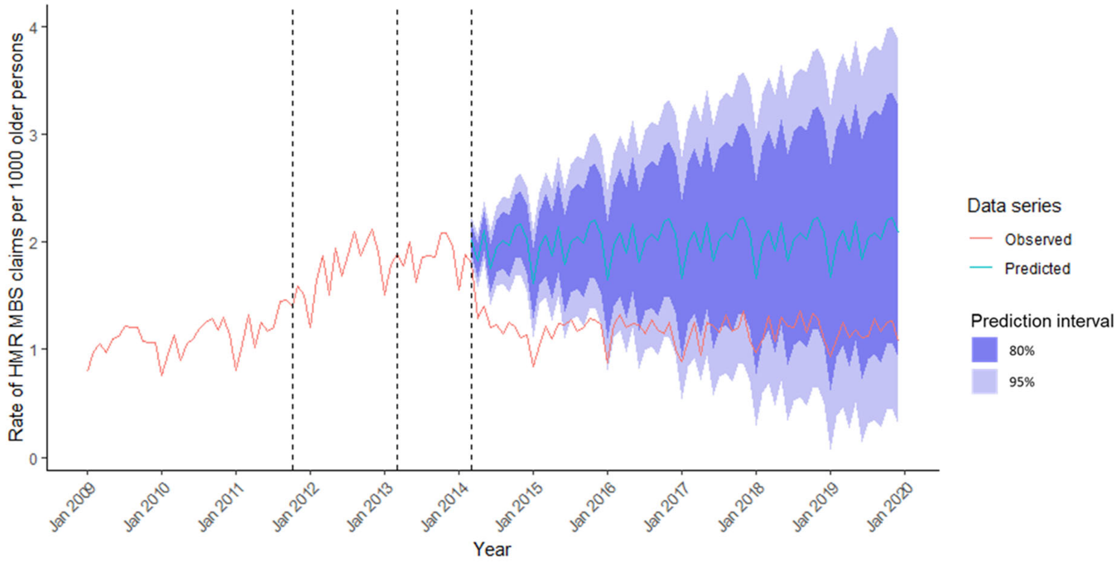

Dashed lines indicate dates of program rule changes

Figure S2. Monthly rate of Residential Medication Management Review (RMMR) claims on the Medicare Benefits Schedule per 1000 older residents of aged care facilities between 2009 and 2019, and counterfactual forecast in the absence of changes to program rules in 2014.

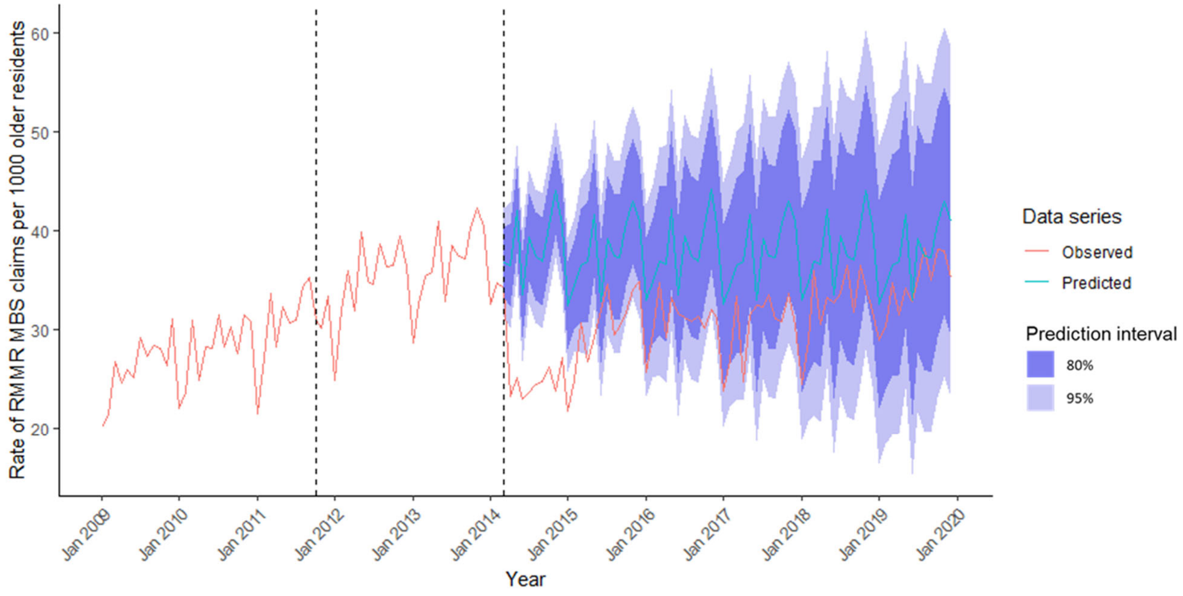

Dashed lines indicate dates of program rule changes
